# Sphingolipid synthesis and scavenging in the intracellular apicomplexan parasite, *Toxoplasma gondii*

**DOI:** 10.1016/j.molbiopara.2012.11.007

**Published:** 2013-01

**Authors:** Steven Pratt, Nilu K. Wansadhipathi-Kannangara, Catherine R. Bruce, John G. Mina, Hosam Shams-Eldin, Josefina Casas, Kentaro Hanada, Ralph T. Schwarz, Sabrina Sonda, Paul W. Denny

**Affiliations:** a Biophysical Sciences Institute, Department of Chemistry and School of Biological Sciences, University Science Laboratories, South Road, Durham DH1 3LE, UK; b School of Medicine and Health, Durham University, Queen's Campus, Stockton-on-Tees TS17 6BH, UK; c Institut für Virologie, Zentrum für Hygiene und Infektionsbiologie, Philipps-Uniersität Marburg, Hans-Meerwein-Strasse, 35043 Marburg, Germany; d Research Unit on Bioactive Molecules (RUBAM), Department of Biomedicinal Biochemistry, Instituto de Química Avanzada de Catalunya, Consejo Superior de Investigaciones Cíentificas (IQAC-CSIC), Barcelona, Spain; e Department of Biochemistry and Cell Biology, National Institute of Infectious Diseases, 1-23-1 Toyama, Shinjuku-ku, Tokyo 162-8640, Japan; f Unité de Glycobiologie Structurale et Fonctionnelle, UMR CNRS/USTL n° 8576 – IFR 118, Université des Sciences et Technologies de Lille, 59655 Villeneuve D’Ascq Cedex, France; g Swiss HPB Center, Pancreatitis Research Laboratory, University Hospital Zurich, Raemistrasse 100, CH-8091 Zürich, Switzerland

**Keywords:** PI, phosphatidylinositol, PC, phosphatidylcholine, PE, phosphatidylethanolamine, IPC, inositol phosphorylceramide, SM, sphingomyelin, CPE, ceramide phosphorylethanolamine, NBD, C_6_-ceramide-*N*-[6-[(7-nitro-2-1,3-benzoxadiazol-4-yl)amino]hexanoyl]-d-*erythro*-sphingosine, CHAPS, 3-[(3-cholamidopropyl) dimethylammonio]-1-propanesulfonate, *Tg*SLS, *Toxoplasma gondii* sphingolipid synthase, MEF, mouse embryonic fibroblasts, *Toxoplasma*, Sphingolipid, Inositol phosphorylceramide synthase, Host–parasite interaction

## Abstract

Sphingolipids are essential components of eukaryotic cell membranes, particularly the plasma membrane, and are involved in a diverse array of signal transduction pathways. Mammals produce sphingomyelin (SM) as the primary complex sphingolipid *via* the well characterised SM synthase. In contrast yeast, plants and some protozoa utilise an evolutionarily related inositol phosphorylceramide (IPC) synthase to synthesise IPC. This activity has no mammalian equivalent and IPC synthase has been proposed as a target for anti-fungals and anti-protozoals. However, detailed knowledge of the sphingolipid biosynthetic pathway of the apicomplexan protozoan parasites was lacking. In this study bioinformatic analyses indicated a single copy orthologue of the putative SM synthase from the apicomplexan *Plasmodium falciparum* (the causative agent of malaria) was a *bona fide* sphingolipid synthase in the related model parasite, *Toxoplasma gondii* (*Tg*SLS). Subsequently, *Tg*SLS was indicated, by complementation of a mutant cell line, to be a functional orthologue of the yeast IPC synthase (AUR1p), demonstrating resistance to the well characterised AUR1p inhibitor aureobasidin A. *In vitro*, recombinant *Tg*SLS exhibited IPC synthase activity and, for the first time, the presence of IPC was demonstrated in *T. gondii* lipid extracts by mass spectrometry. Furthermore, host sphingolipid biosynthesis was indicated to influence, but be non-essential for, *T. gondii* proliferation, suggesting that whilst scavenging does take place *de novo* sphingolipid synthesis may be important for parasitism.

## Graphical abstract

**Graphical abstract:**

**Figure:**
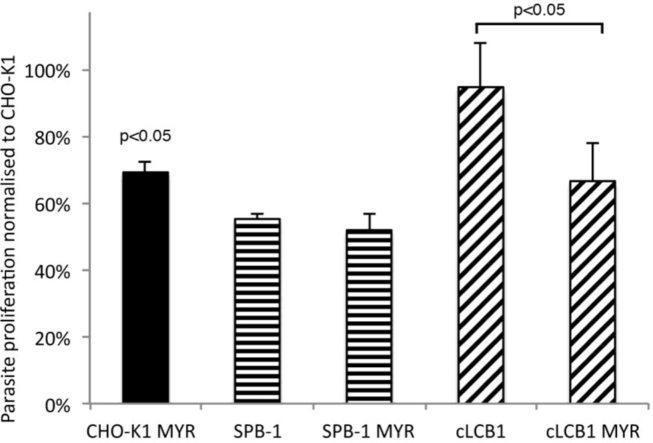

**Highlights:**

► Identification and characterisation of *Toxoplasma* sphingolipid synthase (*Tg*SLS). ► Demonstration of *Tg*SLS inositol phosphorylceramide synthase activity. ► Identification of inositol phosphorylceramide in *Toxoplasma* extracts. ► Delineation of role of host sphingolipid biosynthesis in *Toxoplasma* proliferation. ► Host biosynthesis non-essential for proliferation, *de novo* synthesis could be key.

## 1 Introduction

*Toxoplasma gondii* is an obligate, intracellular protozoan parasite, which is able to invade and colonise a wide variety of nucleated vertebrate cells. It is a member of the Apicomplexa, a diverse phylum including important pathogens of humans and domestic animals such as *Plasmodium* (the causative agent of malaria), *Cryptosporidium* (diarrhoea), *Eimeria* (coccidiosis in poultry) and *Theileria* (East Coast Fever in cattle). *Toxoplasma* has emerged as an opportunistic pathogen and toxoplasmosis is an important disease in the immunocompromised, particularly AIDS patients, those receiving anti-cancer chemotherapy and organ transplant recipients [1]. *Toxoplasma* infection *in utero* is also a significant cause of congenital defects in humans [1] and spontaneous abortion in economically important domestic animals [2].

Sphingolipids are amphipathic lipids comprising sphingosine as the basic building unit. More complex sphingolipids consist of a sphingosine backbone *N*-acylated with a long-chain fatty acid (*i.e.* ceramide) and substituted with a head group moiety (*e.g.* sphingomyelin, glucosylceramide and ceramide-1-phosphate) [3]. Ceramide is a sphingolipid that functions as a secondary messenger in ubiquitous, evolutionarily conserved, signalling mechanisms [4]. Complex sphingolipids are major components of the outer leaflet of eukaryotic plasma membranes that are thought to be involved, together with sterols, in the formation of micro-domains known as lipid rafts. These rafts have been proposed to function in a diverse array of processes from the polarised trafficking of lipid-modified proteins, to the assembly and activation of signal transduction complexes [5]. In the apicomplexan *Plasmodium* species, sphingolipid-enriched lipid rafts have been implicated in the interaction of the parasite with the host erythrocyte through the trafficking of both host and parasite glycosylphosphatidylinositol (GPI) anchored proteins [6]. In addition, it has been demonstrated, by the incorporation of tritiated serine, that both *Plasmodium falciparum* and *T. gondii* synthesise sphingolipids *de novo* [7,8]. Like mammals, *P. falciparum* synthesises the complex phosphosphingolipid sphingomyelin (SM) [9–11] and an orthologue of the mammalian enzyme, SM synthase, has been identified from the genome database [12]. *T. gondii* has also been indicated to synthesise SM, although at relatively low levels compared to glycosphingolipids [8], and the presence of this species has subsequently been confirmed using mass spectrometry [13]. However, the enzyme responsible for any SM synthase activity has remained unidentified in *T. gondii*, and uncharacterised in any apicomplexan. Furthermore, it has also been reported that the parasites harbour relatively high quantities of ceramide phosphorylethanolamine (CPE), a non-abundant species in mammalian cells [13]. In addition, the synthesis of the non-mammalian phosphosphingolipid, inositol phosphorylceramide (IPC), has also been reported in *T. gondii* [14]. Importantly, the biosynthetic enzyme, IPC synthase, has been validated as a drug target in both the fungi and the kinetoplastid protozoa [15–18], and its inhibition by the anti-fungal aureobasidin A has been proposed in *T. gondii* [14].

Notably, in addition to *de novo* synthesis, intracellular parasites such as *T. gondii* may scavenge sphingolipids or their precursors from the host cell [19]. Indeed it has been suggested that the CPE (and SM) found in intracellular tachyzoites forms may result from the concentration of non-abundant host-derived lipid [13]. Within the host cell *T. gondii* resides within a specialised parasitophorous vacuole (PV) formed immediately after invasion and delineated by the PV membrane (PVM) [20]. Although the PV resists fusion with host organelles it does demonstrate an intimate, high affinity association with the ER and mitochondrion [21], the latter facilitating the scavenging of host lipoic acid [22]. Furthermore, recent work has indicated that host-derived lipid is the primary contributor to the intravacuolar network that fills the lumen of the PV [23]. *Toxoplasma* scavenges a variety of fatty acids and lipids from the host, including phospholipids and cholesterol, some of which are further metabolised by the parasite [24,25]. The mechanism of lipid scavenging is unclear, although current data argue against passive diffusion, acquisition on invasion [25] and (at least in the case of cholesterol) vesicular trafficking [24]. It has been proposed that the transport of cholesterol to the PV could be mediated *via* a protein carrier [24], and the possibility of direct inter-organelle transfer of lipids between the closely associated PVM and host ER and mitochondrial membranes has been evoked [22,25]. The balance between *de novo* synthesised and scavenged lipid is unclear, however when host phosphatidylcholine (PC) levels are restricted it is likely that the parasites scavenge choline and synthesise PC *de novo* [25].

To begin to understand the role of both *de novo* synthesis and scavenging of sphingolipid for *T. gondii*, we aimed to begin characterisation of the little understood parasite biosynthetic pathway and investigate the requirement, if any, for host sphingolipid. To these ends we herein report the identification and functional characterisation of a key enzyme in *T. gondii* sphingolipid synthesis that may represent a novel drug target and, in addition, show the delineation of the role of host biosynthesis in parasite proliferation.

## 2 Materials and methods

### 2.1 Selection, sequence analyses and cloning of candidate sphingolipid synthase

The *T. gondii* genome database (www.toxodb.org) was interrogated (Gish, 1996–2001) (http://blast.wustl.edu) with the two candidate sphingolipid synthase coding sequences previously identified from the genome database of the malaria parasite *P. falciparum* (plasmodb.org) [12]. A single sequence orthologue was identified, *Tg*SLS accession number TGME49_046490, corresponding to the entry previously identified [13]. Sequence alignments were made using ClustalW [26] and phylogenetic analyses performed on the edited alignments using Maximum Parsimony, Protein Distance (PHYLIP Phylogeny Inference Package, version 3.5c) and Maximum Likelihood [27]. The candidate *Tg*SLS open reading frame was amplified from genomic *T. gondii* DNA using *Pfu* polymerase (Promega) and the primer pair 5′TgSLSEcoRI (cgcgaattcATGCCCAGAACAGAGATG) and 3′*Tg*SLS*HindIII (cccaagcttTTAGAGTCCCTCGATGGCGCGAACGAT). Cloning sites shown in lower case, with coding sequence in upper case. The product was purified, digested and cloned into the yeast expression vector pRS426MET25 creating pRS426 *Tg*SLS.

### 2.2 Functional complementation of auxotrophic yeast AUR1 mutant

pRS426 *Tg*SLS, together with pRS426 AUR1 and empty vector, were used to transform the YPH499-HIS-GAL-AUR1 *Saccharomyces cerevisiae* strain [28]. Transformants were selected on non-permissive SD-HIS-URA medium (0.17% Bacto yeast nitrogen base, 0.5% ammonium sulphate and 2% dextrose) or permissive SGR-HIS-URA medium (0.17% Bacto yeast nitrogen base, 0.5% ammonium sulphate, 4% galactose and 2% raffinose) containing the appropriate nutritional supplements at 30 °C.

### 2.3 *In vitro* assay of *Tg*SLS activity

Microsomal membranes from exponentially growing YPH499-HIS-GAL-AUR1 pRS426 *Tg*SLS or pRS426 AUR1 in SD-HIS-URA were prepared and the isolated membrane fraction re-suspended in storage buffer (50 mM Tris–HCl pH 7.4, 20% (v/v) glycerol, 5 mM MgCl_2_) with Complete^®^ EDTA-free Protease Inhibitor Cocktail (Roche Applied Science) at a protein concentration of 10 mg/ml as described previously [18]. Microsomal membranes were subsequently washed in 40 mM CHAPS (4 °C, 60 min), isolated by centrifugation (150,000 × *g*, 4 °C and 100 min), re-suspended in storage buffer at 10 mg/ml and stored at −80 °C until use. The assay mix contained 1 mM donor substrate (bovine liver PI, PC or PE, Avanti Polar Lipids), 20 μg microsome prep, 100 mM Tris–HCl, 10 mM EDTA, 6 mg/ml BSA and 5 μM NBD C_6_-ceramide [19]. Following incubation at 30 °C for 60 min the reaction was quenched by the addition of 150 μl of chloroform:methanol:water (10:10:3) and lipids separated and analysed as above. For inhibition experiments the reaction mix was pre-incubated for 30 min with appropriate quantities of aureobasidin A (Takara Bio Inc.) before the addition of NBD C_6_-ceramide.

### 2.4 Agar diffusion assay

YPH499-HIS-GAL-AUR1 complemented with *TgSLS* or AUR1 were assayed for susceptibility to aureobasidin A and myriocin (Sigma) as previously described [28]. Briefly, 2.4 OD_600_ units of logarithmically dividing cells were embedded in 15 ml of SD-HIS-URA with 0.8% agarose on 100 mm^2^ square Petri dishes (Sarstedt). Inhibitors were applied in DMSO at the concentrations indicated and the dishes incubated at 30 °C.

### 2.5 Mass spectrometry of *T. gondii* inositol phosphorylceramide

*T. gondii* (strain RH) were harvested from infected MEF host cells by passage through a 26 gauge needle and purified by separation on Sephadex-G25 columns (Amersham). Following PBS wash, the parasites were transferred to glass vials. Sphingolipid extracts were prepared as described [29] and analysed. The liquid chromatography-mass spectrometer consisted of a Waters Aquity UPLC system connected to a Waters LCT Premier orthogonal accelerated time of flight mass spectrometer (Waters, Millford, MA), operated in negative electrospray ionisation mode. Mass accuracy and reproducibility were maintained by using an independent reference spray by the LockSpray interference. The analytical column was a 100 mm × 2.1 mm i.d., 1.7 mm C8 Acquity UPLC BEH (Waters). The two mobile phases were A: methanol:water:formic acid (74:25:1); B: methanol:formic acid (99:1), both also contained 5 mM ammonium formate. A linear gradient was programmed as follows: 0.0 min: 80% B; 3 min: 90% B; 6 min: 90% B; 15 min: 99% B; 18 min: 99% B; 20 min: 80% B. The flow rate was 0.3 ml/min. Positive identification of all sphingolipid species was based on the accurate mass measurement with an error <5 ppm and their LC retention time.

### 2.6 Cell culture

*T. gondii* (strain RH, TATi-1; a kind gift from Prof Dominique Soldati-Favre, University of Geneva) were maintained in Vero cells grown in DMEM supplemented with 10% foetal bovine serum (FBS) at 37 °C and 5% CO_2_. Parasites were separated from host cell material by filtration through 3 and 5 μm polycarbonate filters (Millipore) after disruption by passage through a 26 gauge needle. Chinese Hamster Ovary (CHO-K1) cells, the derived temperature sensitive serine palmitoyl transferase mutant SPB-1, plus SPB-1 complemented with cLCB-1 [30] were grown in Ham's F-12 media (Sigma–Aldrich) supplemented with 10% FBS (Gibco) at 37 °C or 33 °C (SPB-1 permissive temperature) with 5% CO_2_.

### 2.7 Suppression of host sphingolipid synthesis

For induction of the temperature-sensitive phenotype 10^5^ cells were seeded into 24-well plates and incubated at 33 °C for 24 h. The medium was then replaced with Ham's F12 medium supplemented with 10% FBS or, to form serum reduced media, 0.1% FBS, 1% Nutridoma-SP (Roche Applied Science), 250 μM oleic acid (Sigma Aldrich) and 5% fatty acid free Bovine Serum Albumen (BSA; Sigma Aldrich). The low-serum medium contains less than 1 μM sphingomyelin [31]. As indicated, myriocin (Sigma Aldrich) was added at this stage. Cells were then incubated at 39 °C for 72 h before *T. gondii*, purified as above, were added at a ratio of 2 parasites to 1 host cell and allowed to invade for 4 h. Subsequently, after washing with PBS, cells were incubated for a further 24 h in normal or serum reduced media before assay. Cell viability under these conditions was confirmed by staining with trypan blue (Sigma Aldrich).

### 2.8 Parasite invasion

*T. gondii* infected cells were fixed with methanol at −20 °C for 10 min, dried and stained with 300 nM DAPI in PBS for 5 min. Visualisation using an Olympus 1X-71 epifluorescent microscope allowed invasion to be scored.

### 2.9 Parasite proliferation

Following the 4 h invasion time, 1.0 μCi of [5,6-^3^H] uracil (GE Healthcare) was added per well and cultures incubated for 24 h. Supernatant was removed and cells then solublised with 250 μl of 1% (w/v) SDS containing 400 μg of unlabelled uracil/ml. 750 μl of 0.3 M TCA was then added and, after a 15 min incubation at 4 °C, precipitates were collected onto glass fibre filters in 96 well plates (Multiscreen HTS, Millipore) using a sampling manifold. The filters were washed twice with 0.3 M TCA and once with 95% ethanol, dried, placed in 10 ml of scintillation cocktail and the incorporated radioactivity then measured (Wallac 1450 MicroBeta TriLux, Perkin Elmer).

## 3 Results and discussion

### 3.1 Identification of the *T. gondii* sphingolipid synthase

**Supplementary Figure 1:**
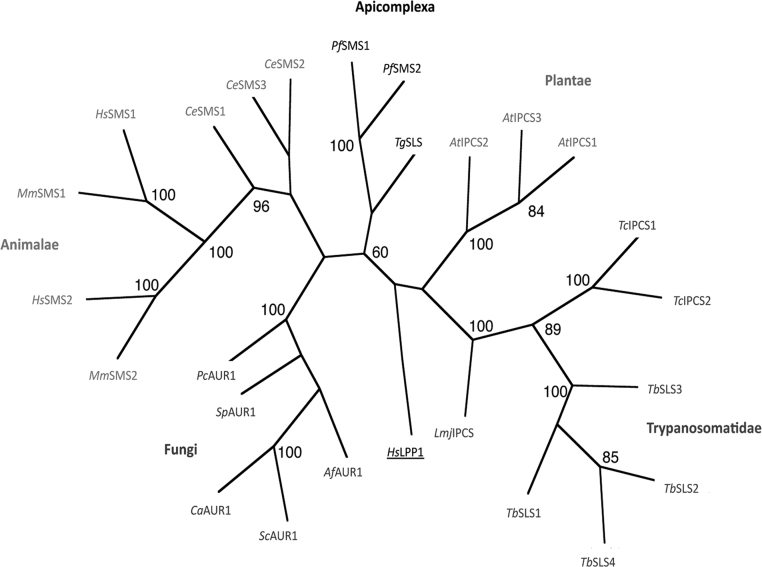
Maximum parsimony analyses of Animalae, Fungi, Trypanosomatidae, Plantae and Apicomplexa sphingolipid synthase predicted amino acid sequences. Bootstrap scores >60 indicated. *Homo sapiens* LPP1 (outgroup) accession number: O14494; *T. gondii* SLS: TGME49_046490; *P. falciparum* SMS1&2: PFF1210w and PFF1215w; *Arabidopsis thaliana* IPCS1-3: At3g54020.1, At2g37940.1, At2g29525.1; *T. brucei* SLS1-4: Tb09.211.1030, Tb09.211.1020, Tb09.211.1010, Tb09.211.1000; *T. cruzi* IPCS1&2: Tc00.1047053506885.124, Tc00.1047053510729.290; *L. major* IPCS: LmjF35.4990; *Aspergillus fumigatus* AUR1p: AAD22750; *Candida albicans* AUR1p: AAB67233; *Pneumocystis carinii* AUR1p: CAH17867; *Saccharomyces cerevisiae* AUR1p: NP_012922; *Schizosaccharomyces pombe* AUR1p: Q10142; *Caenorhabditis elegans* SMS1-3: Q9U3D4, AAA82341, AAK84597; *Homo sapiens* SMS1&2: AB154421, Q8NHU3; *Mus musculus* SMS1&2: Q8VCQ6, Q9D4B1.

**Fig. 1:**
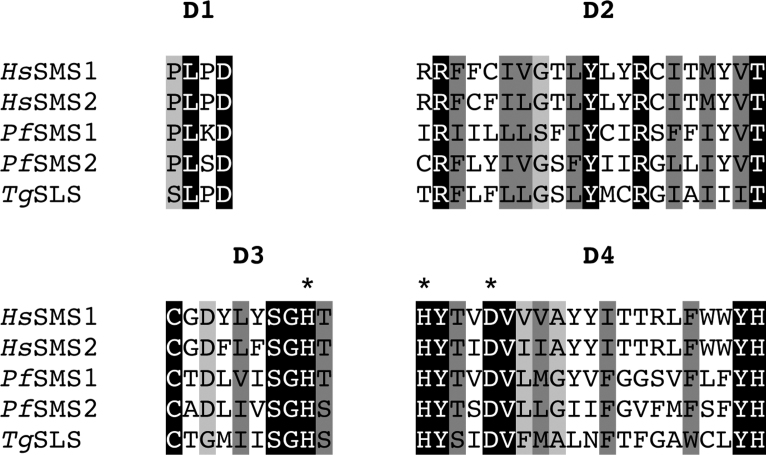
Identification of a candidate sphingolipid synthase from *Toxoplasma gondii* (*Tg*SLS). Protein sequence alignment of D1, D2, D3 and D4 from *Hs*SMS1 and 2, *Pf*SMS1 and 2 and *Tg*SLS. The positions highlighted in black are fully conserved; those in dark grey show conservation of strongly similar groups; those in light grey show conservation of weakly similar groups. The 3 residues of the predicted catalytic triad within D3 and D4 are designated by *.

Although *T. gondii* has been demonstrated to synthesise complex sphingolipids *de novo* [7,8], the identity of the enzymes responsible for this have remained unclear. However, it has been shown, by incorporation of tritiated inositol, that *T. gondii* tachyzoites synthesise the non-mammalian, complex phosphosphingolipid, inositol phosphorylceramide (IPC) [14]. In addition, the synthesis of glycosphingolipids and sphingomyelin (SM), the predominant mammalian phosphospingolipid, have been similarly demonstrated [7,8]. Previous bioinformatic analyses have identified 2 orthologues of the mammalian SM synthase encoded by *P. falciparum, Pf*SMS1 and 2 [12], an apicomplexan known to synthesise this sphingolipid species [9], and a single putative orthologue in the *T. gondii* genome [13]. This *T. gondii* predicted protein demonstrated only 28% and 27% identity to *Pf*SMS1 and 2 respectively. However, further analyses of the predicted amino acid sequence identified the canonical SM synthase domains (D1-4) [12] (Fig. 1), strongly suggesting that this predicted protein is evolutionarily and functionally related to this class of enzymes. Phylogenetic analyses using the Maximum Parsimony algorithm (PHYLIP Phylogeny Inference Package, version 3.5c), of aligned amino acid sequence (*Tg*SLS amino acids 92–362) including the active site residues defined by D3 and D4, supported this hypothesis and indicated that the apicomplexan sphingolipid (SL) synthases form a new group in a wider enzyme family that includes both SM and IPC synthases (Figure S1) [32]. However, further analyses using Maximum Likelihood and Protein Distance (PHYLIP) algorithms (data not shown) failed to support this result demonstrating the surprising divergence of *Tg*SLS with respect to the other sphingolipid synthases, including *Pf*SMS1 and 2.

**Fig. 2:**
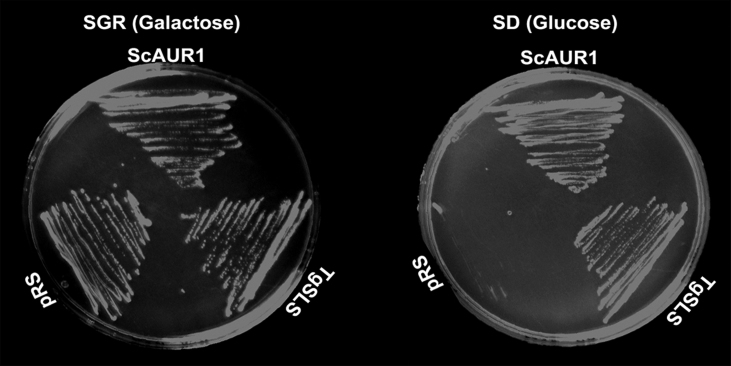
*Tg*SLS complements a yeast AUR1p auxotrophic mutant. The growth of YPH499 HIS-GAL-AUR1 transformed with pRS426 *Tg*SLS, a positive control (pRS426 *Sc*AUR1) and a negative control (empty pRS426, pRS) was supported on permissive SGR media (galactose as carbon source). In contrast, only pRS426 *Tg*SLS and the positive control could grow on non-permissive SD media (glucose as carbon source).

No functional analyses of these apicomplexan enzymes has previously been undertaken. Therefore, the open reading frame of the putative *Toxoplasma* SL synthase (*Tg*SLS) was cloned into an URA3 selectable expression vector creating pRS426 *Tg*SLS. Ectopic expression from this vector was subsequently demonstrated to restore the grow of YPH499-HIS-GAL-AUR1, the previously constructed AUR1 auxotrophic mutant [27], in non-permissive glucose containing media (Fig. 2). These data indicated that *Tg*SLS is a functional orthologue of the yeast IPC synthase, AUR1p [33].

### 3.2 The functionality of the *T. gondii* sphingolipid synthase

**Supplementary Figure 2:**
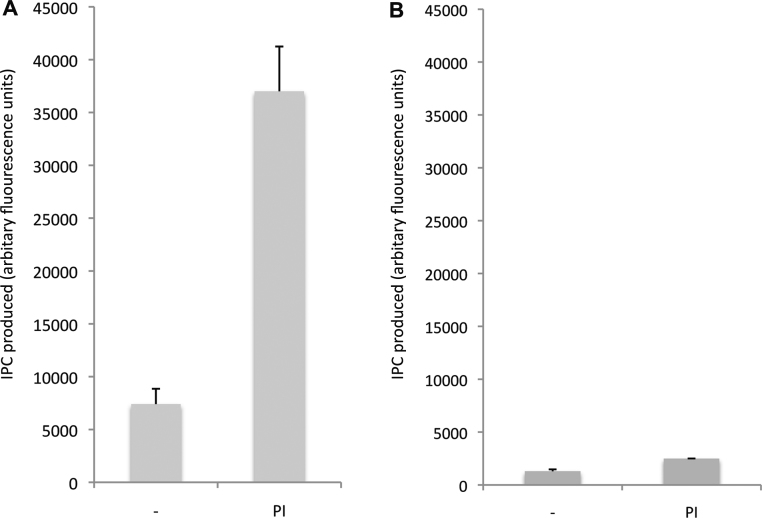
(A) *In vitro* assay of *Tg*SLS with no donor substrate (−) or PI demonstrated that IPC synthase activity was increased approximately 5-fold in the presence of this donor substrate; (B) In contrast, in an equivalent assay *Sc*AUR1 was non-responsive with the bovine PI utilised. AFU – arbitrary fluorescence units. Mean of 3 independent experiments, standard deviation indicated.

**Fig. 3:**
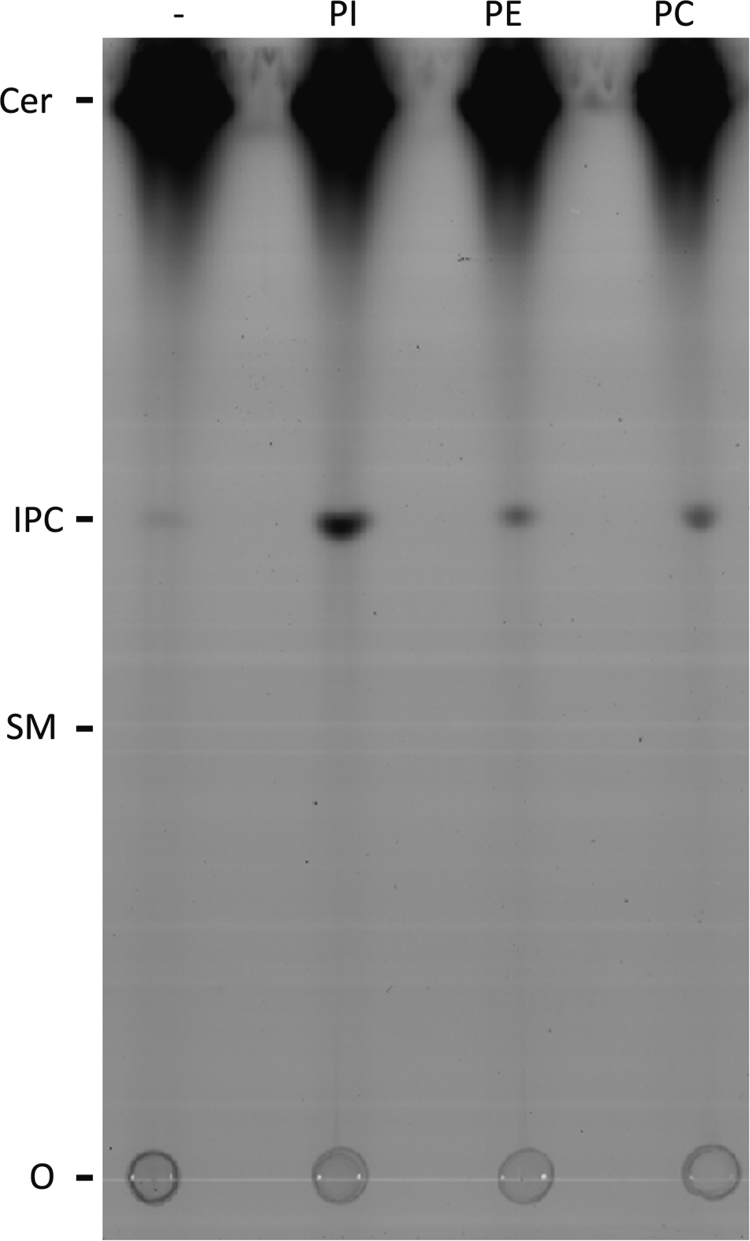
*Tg*SLS functions as an inositol phosphorylceramide synthase. HPTLC fractionation of lipids after reaction of CHAPS-washed *Tg*SLS extract with acceptor substrate NBD-C_6_-ceramide and either no donor substrate (−) or phosphatidylinositol (PI), phosphatidylethanolamine (PE) or phosphatidylcholine (PC). Only the addition of PI led to a significant increase in the product formation, a species migrating with inositol phosphorylceramide (IPC). A representative image, O was the origin, ceramide (Cer) migrated at the front, markers from extracts of NBD-C_6_-ceramide labelled yeast (NBD-IPC) and mammalian Vero cells (NBD-SM).

Given the evidence that *Toxoplasma* synthesise SM [7], IPC [14] and, perhaps, CPE [13] it was necessary to ascertain the functionality of *Tg*SLS. To this end, microsomes were prepared from YPH499-HIS-GAL-AUR1 pRS426 *Tg*SLS and formatted into an *in vitro* assay as previously described for other enzymes of this class [17,18,32]. The detergent wash in the preparation of the microsomes in this process removes lipids and therefore renders the enzyme largely dependent on the addition of exogenous substrate, the labelled acceptor substrate NBD-C_6_-ceramide and the donor substrate, phosphatidylinositol (for IPC synthesis) or phosphatidylcholine (for SM) [17]. *Tg*SLS clearly synthesises a product migrating with IPC on the addition of exogenous PI, but no evidence for the utilisation of PC for SM synthesis or phosphatidylethanolamine (PE) for CPE synthesis was seen under the conditions employed (Fig. 3). The *Toxoplasma* enzyme clearly functions as an IPC synthase, an activity that is increased approximately 5-fold on the addition of the donor substrate PI to the assay (Figure S2A). There was also a small increase in IPC signal on the addition of both PC and PE. This small effect was consistent across four repeats and could be due to these lipids altering the composition of the micelles formed after the CHAPS wash, perhaps leading to increased availability of the remaining endogenous PI. In contrast, the yeast IPC synthase, AUR1p, is non-responsive to the mammalian PI employed in this assay system (Figure S2B) [18].

### 3.3 Inositol phosphorylceramide in *T. gondii*

**Fig. 4:**
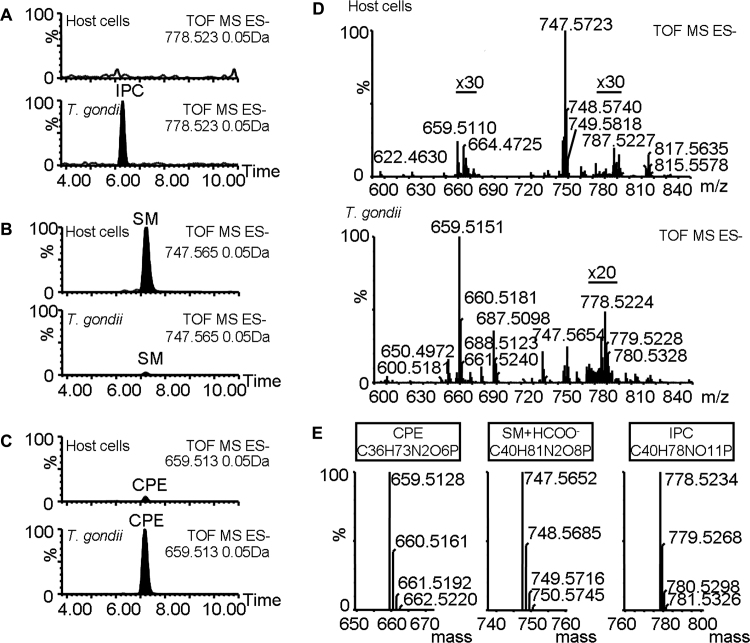
Inositol phosphorylceramide identification in *Toxoplasma gondii* lipid extracts. (A) Selected negative ion m/z 778.5234 (inositol phosphorylceramide, *N*-hexadecanoyl (N-C16) species, IPC); (B) selected negative ion m/z 747.5652 (sphingomyelin, *N*-hexadecanoyl (N-C16) species, SM); and (C) selected negative ion *m*/*z* 659.5128 (ceramide phosphorylethanolamine, *N*-hexadecanoyl (N-C16) species, CPE) UPLC–TOF chromatograms of mouse embryonic fibroblast (MEF) host cells and *T. gondii* lipid extracts. (D) Partial mass spectra (from 590 to 850 amu) corresponding to the 6 and 7.8 min range of a representative chromatogram obtained by UPLC/TOF-ESI(−) analysis of lipid extracts of host cells and *T. gondii*. Regions amplified (20× or 30×) as indicated. (E) Theoretical mass spectral pattern for the molecular ion region showing an (M–H)-ion for CPE and IPC and (M + HCOO^−^) for SM. CPE and SM were found in both samples, whereas IPC was only identified in *T. gondii* extracts.

Lipidomic analyses have identified the presence of SM, CPE, but not IPC in *Toxoplasma* [13]. Therefore, in order to investigate whether the parasite harbours this non-mammalian sphingolipid species analyses by liquid chromatography–mass spectrometry (LC–MS) [34] of lipids extracted from isolated *T. gondii* and host mouse embryonic fibroblast (MEF) cells were performed (Fig. 4). The unambiguous identification of IPC was achieved by accurate mass determination by high resolution time-of-flight (TOF) mass spectrometry. A selected negative ion UPLC–TOF chromatogram, corresponding to the deprotonated C16-IPC species, of the *T. gondii* lipid extract exhibited a peak absent in the mammalian host cells (Fig. 4A). In contrast, selected negative ions corresponding to the formic acid adduct of SM (Fig. 4B) and deprotonated CPE (Fig. 4C) were detected in both host and parasite extracts, with CPE appearing enriched in *T. gondii* as previously recorded [13]. The mass spectrum of these peaks (Fig. 4D) showed an accurate mass measurement with errors with respect to the theoretical spectra (Fig. 4E), of 0.3 ppm and 3.5 ppm for SM and CPE, and 0.6 ppm for IPC. In addition, they demonstrated very similar isotopic patterns. These data demonstrate, for the first time, the presence of non-mammalian IPC (C18:1/C16:0) in *T. gondii* and strongly suggest that the IPC synthase activity of *Tg*SLS seen *in vitro* above is also evident *in vivo.*

### 3.4 Inositol phosphorylceramide synthase as a drug target in *T. gondii*

**Fig. 5:**
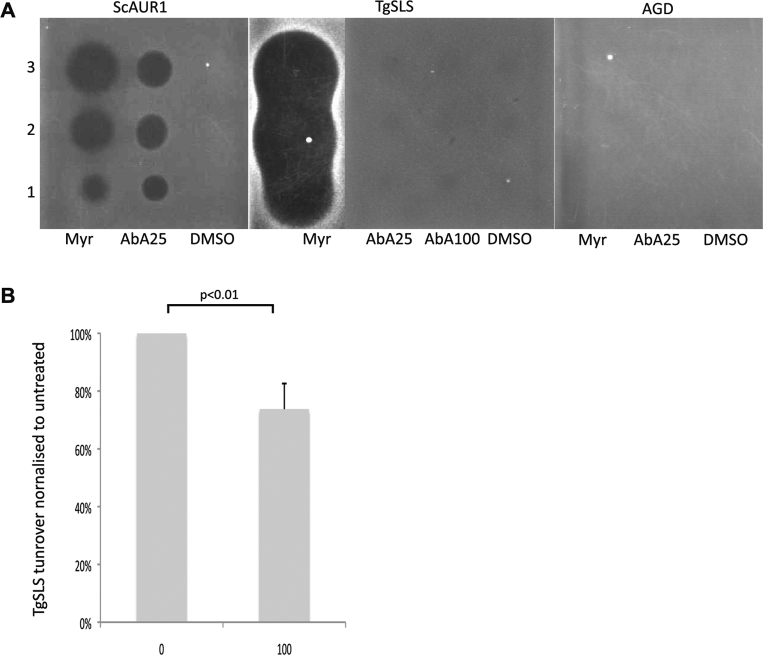
*Tg*SLS sensitivity to a verified yeast AUR1p inhibitor. (A) Agar diffusion assay of YPH499 HIS-GAL-AUR1 complemented yeast showed that, as expected, *Sc*AUR1 complemented yeast were sensitive to myriocin at 1 mM (Myr) and aureobasidin A at 25 μM (AbA25). In contrast, *Tg*SLS complemented yeast were resistant to AbA at 25 μM and 100 μM (AbA100), but hyper-sensitive to myriocin (1 mM) as evidenced by large zones of exclusion. AGD, the sphingolipid bypass mutant yeast lacking functional SPT and AUR1, acted as the negative control. DMSO was the vehicle control. (B) *In vitro* assay of the inhibitory effect of aureobasidin A (AbA) on *Tg*SLS demonstrated that the *Toxoplasma* enzyme is only marginally, but significantly (*p* < 0.01), sensitive to the drug at a high concentration (100 μM). Fluorescence intensity of IPC was established following fractionation by HPTLC and normalised with respect to an untreated control. Mean of 3 independent experiments, standard deviation indicated.

The, then uncharacterised, *Toxoplasma* IPC synthase activity has previously been proposed as a drug target based on the efficacy of the potent, well-characterised fungal IPC synthase inhibitor aureobasidin A (AbA) against *T. gondii* within infected host cells [14]. To test this hypothesis we chose to analyse the sensitivity of YPH499-HIS-GAL-AUR1 pRS426 *Tg*SLS to AbA by diffusion assay (Fig. 5A). AbA sensitive YPH499-HIS-GAL-AUR1 pRS426 AUR1 was used as a positive control and the sphingolipid bypass mutant AGD as a negative control. AGD is able to grow without synthesising sphingolipids and therefore is able to tolerate loss-of-function mutations in both serine palmitoyltransferase and IPC synthase [33]. These results demonstrated that *Tg*SLS (the *Toxoplasma* IPC synthase) conferred AbA resistance to the yeast. The hyper-sensitivity to myriocin (an inhibitor of an upstream step in sphingolipid biosynthesis mediated by serine palmitoyltransferase) resembled that seen in the same mutant yeast complemented by the *Leishmania major* IPC synthase [28]. The reasons for this are unknown but perhaps reflect a sub-optimum functionality of the protozoan sphingolipid synthases in the yeast making the complemented lines more sensitive to upstream inhibition of sphingolipid biosynthesis.

To further investigate any inhibition of *Toxoplasma* IPC synthase activity by AbA the microsomal assay described above was employed (Fig. 5B). Small, but statistically significant, inhibition of enzyme turnover was noted at 100 μM. However, in contrast it should be noted that the aureobasidin A is a tight binding inhibitor of the yeast IPC synthase (AUR1p) with a reported IC_50_ of 0.2 nM [33]. Together, these data demonstrate that *Tg*SLS is largely resistant to AbA and, as such, resembles the characterised, AbA-resistant IPC synthase from the kinetoplastid protozoan parasite, *L. major* [28].

Therefore, *Tg*SLS is not the target for aureobasidin A in *T. gondii* and the previously reported efficacy [14] may be due to unidentified off target host effects as hypothesised for the kinetoplastid protozoa *T. cruzi* [35].

### 3.5 The role of host sphingolipid biosynthesis in *T. gondii* proliferation

As demonstrated above and previously [7] *T. gondii* have the ability to synthesise sphingolipids *de novo*. However, as an intracellular parasite *T. gondii* may also utilise host sphingolipids. In order to unravel the roles of host synthesis in parasite proliferation, Chinese Hamster Ovary (CHO) cells with a temperature sensitive serine palmitoyltransferase (SPT) were utilised as host cells [30,31]. SPT is the first and rate-limiting enzyme in eukaryotic sphingolipid biosynthesis and in this cell line, SPB-1, the LCB1 subunit of this heterodimeric enzyme is thermolabile. At the non-permissive temperature virtually no SPT activity is detectable and sphingolipids are depleted when the SPB-1 cells are grown with minimal sera [36]. This phenotype is reversed by stable transfection of cDNA encoding cLCB1 [36]. Like *T. gondii*, the bacterial pathogen *Chlamydia trachomatis* (the causative agent of trachoma) resides within a non-fusagenic inclusion. Within SPB-1 cells *C. trachomatis* are completely unable to replicate at the non-permissive temperature, demonstrating that host sphingolipid biosynthesis is essential for intracellular growth of this pathogen [37] which acquires newly synthesised SM *via* the host exocytic pathway [38–41].

**Fig. 6:**
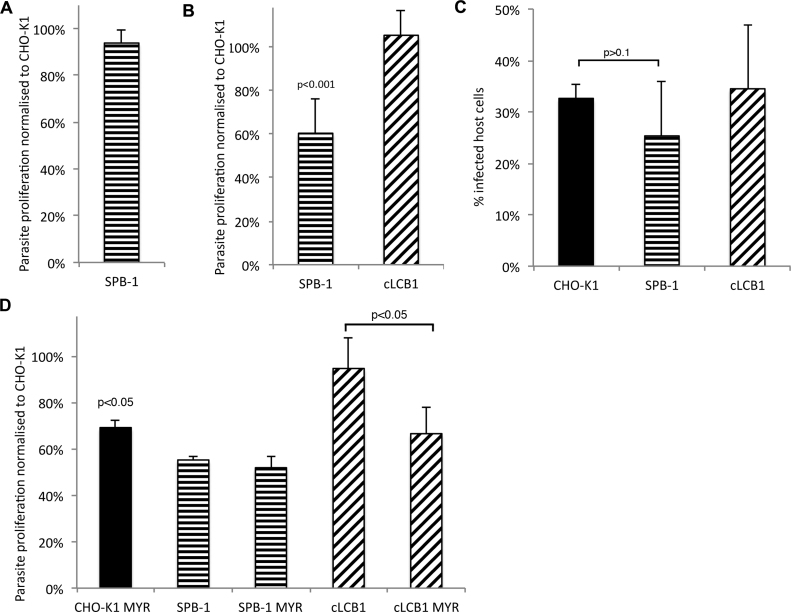
Analyses of the role of host serine palmitoyltransferase (SPT) in *Toxoplasma gondii* proliferation and invasion. Cells were cultured in 10% FCS (A) or in serum-reduced media (B) at the non-permissive temperature (39 °C). In the presence of complete media, *T. gondii* proliferation was the same in wild type (CHO-K1) and SPT-compromised (SPB-1) host cells (A). However, in serum-reduced media proliferation was significantly (*p* < 0.001) decreased in SPB-1 cells compared to the control (CHO-K1 and SPB-1 cLCB1) lines (B). All results normalised with respect to proliferation in parental CHO-K1 cells. Analyses of 3 independent experiments performed in triplicate, standard deviation indicated. This effect was not due to significant differential invasion of the host cell lines (*p* > 0.1; C). Analyses of 3 independent experiments, standard deviation indicated. To analyse the effect of chemical inhibition of SPT, cells were cultured in serum-reduced media at the non-permissive temperature (39 °C) in the presence or absence of the inhibitor, myriocin (D). Myriocin treatment reduced *Toxoplasma* proliferation (*p* < 0.05) in wild type (CHO-K1) cells to similar levels to those seen in untreated SPT-compromised (SPB-1) host cells. The compound had no effect on proliferation in SPB-1 cells but exerted a similar effect to wild type in complemented mutant cells (SPB-cLCB1; *p* < 0.05). All results normalised with respect to proliferation in parental CHO-K1 cells. Analyses of experiments performed in triplicate, standard deviation indicated.

Utilising the same conditions employed in study of *C. trachomatis* replication [37], the proliferation of *T. gondii* in SPB-1 CHO cells was investigated. Briefly, before invasion host cells (both SPB-1 and controls) were incubated for 72 h at the non-permissive temperature (39 °C) in either complete or serum-reduced media. In the same media the cells were infected with isolated *T. gondii* as described in Section 2, and proliferation established by measuring [^3^H]-uracil incorporation as previously described [42] (Fig. 6A and B). It was clear that under the non-permissive conditions but in complete media the parasites replicated as well in the SPT deficient SPB-1 cells as they did in the wild type parental line (Fig. 6A). In contrast, with serum-reduced media *T. gondii* proliferation in the ts mutant cells was significantly reduced compared with the wild type control (*p* < 0.001; Fig. 6B). Notably, this effect was rescued in SPB-1 cells complemented by the expression LCB1 from stably transfected cDNA. Furthermore, these results are not due to differential *T. gondii* invasion as, under these conditions, there was no significant difference (*p* > 0.1) in the invasion rate between any of the cell lines employed (Fig. 6C). SPT can also be specifically inhibited by the natural product sphingosine analogue, myriocin [43]. Importantly, this induced a similar decrease in *T. gondii* proliferation in wild type CHO cells to that seen in the SPB-1 mutant. No effect was observed on treatment of the SPB-1 cells in which SPT activity was already suppressed, indicating that the effect seen in wild type cells was due to inhibition of host SPT and not the uncharacterised parasite orthologue (Fig. 6D). This may be due to the *Toxoplasma* SPT being either resistant or inaccessible to myriocin. Alternatively modulation of parasite SPT activity may have only a limited effect on proliferation. However, whilst host sphingolipid biosynthesis clearly plays a role in parasite replication, unlike for the bacterium *C. trachomatis* [37], it is non-essential. Furthermore, the addition of serum to the media negated any effect, indicating that scavenging from the extracellular milieu can compensate for the lack of host SPT activity. Notably, previous studies of *T. gondii* development within enucleated cells did not record any significant differences in parasite growth compared to nucleated controls [44]. Taken together these results indicate that host synthesis is not immediately critical for the ability of the parasite to grow, although prolonged depletion of sphingolipids (both synthesised and exogenous for 96 h) does influence proliferation (an approximate 40% reduction). This contrasts with the intra-cellular kinetoplastid, protozoan parasite *Leishmania mexicana* where suppression of host sphingolipid synthesis and depletion of extracellular lipid showed no effect on parasite proliferation [45].

## 4 Conclusion

*T. gondii* is an important cause of disease in humans and domestic animals and a model apicomplexan pathogen [46]. The essential, eukaryotic sphingolipids have been implicated in the interaction of the related apicomplexan *P. falciparum* with its anucleate host cell, the erythrocyte [6]. In this study we utilised the ability of *T. gondii* to invade and colonise a wide range of nucleated vertebrate cells to examine the role host sphingolipid synthesis in invasion and proliferation. Genetic and chemical inhibition of the host pathway, coupled with depletion of media lipid, inhibited parasite proliferation (but not invasion). However, in contrast to the bacterium *C. trachomatis* [37], *T. gondii* was still able to replicate under these conditions. Notably, while auxotrophic for sterols [24], *T. gondii* have maintained the ability to synthesise sphingolipids *de novo* [7]. It could be hypothesised that this allows the parasite to survive and replicate in conditions of greatly reduced host sphingolipid biosynthesis. However, the protozoan biosynthetic pathway and the identity of the enzymes that constitute it remained relatively uninvestigated. In this study we showed that the identified *T. gondii* SL synthase demonstrated IPC synthase activity *in vitro*, and that this non-mammalian lipid species is detectable by mass spectrometry in parasite extracts. Given the status of the fungal AUR1p and kinetoplastid IPC synthases as promising drug targets [3], the identification of the *T. gondii* orthologue opens up the possibility of targeting this enzyme with novel anti-protozoals. However, the ability of the parasite to scavenge sphingolipid from the host may complicate the viability of *Tg*SLS as a pharmaceutical target and clearly the balance between *de novo* synthesis and scavenging merits further investigation.
